# Social Models Provide a Norm of Appropriate Food Intake for Young Women

**DOI:** 10.1371/journal.pone.0079268

**Published:** 2013-11-13

**Authors:** Lenny R. Vartanian, Nicole Sokol, C. Peter Herman, Janet Polivy

**Affiliations:** 1 School of Psychology, The University of New South Wales, Sydney, New South Wales, Australia; 2 Department of Psychology, University of Toronto, Toronto, Ontario, Canada; University of Queensland, Australia

## Abstract

It is often assumed that social models influence people’s eating behavior by providing a norm of appropriate food intake, but this hypothesis has not been directly tested. In three experiments, female participants were exposed to a low-intake model, a high-intake model, or no model (control condition). Experiments 1 and 2 used a remote-confederate manipulation and were conducted in the context of a cookie taste test. Experiment 3 used a live confederate and was conducted in the context of a task during which participants were given incidental access to food. Participants also rated the extent to which their food intake was influenced by a variety of factors (e.g., hunger, taste, how much others ate). In all three experiments, participants in the low-intake conditions ate less than did participants in the high-intake conditions, and also reported a lower perceived norm of appropriate intake. Furthermore, perceived norms of appropriate intake mediated the effects of the social model on participants’ food intake. Despite the observed effects of the social models, participants were much more likely to indicate that their food intake was influenced by taste and hunger than by the behavior of the social models. Thus, social models appear to influence food intake by providing a norm of appropriate eating behavior, but people may be unaware of the influence of a social model on their behavior.

## Introduction

Food is often consumed in the presence of others, be it a family meal, a lunch meeting, or a weekend barbeque. In these situations, there are a number of ways in which the behavior (or even the mere presence) of other people can influence one’s own food intake. One of the most robust social influences on people’s food intake is modeling. In essence, people tend to follow the lead of an eating companion, eating more when the companion eats more, and eating less when the companion eats less [1]. The effects of modeling of food intake have been shown in correlational studies (e.g., [2]) as well as in experimental studies (e.g., [3], [4]). An intriguing finding from the literature on modeling of food intake is the variety of conditions under which these effects are observed. Modeling is observed with snack foods [5] and during meals [6], among dieters and non-dieters [7], among children [8], and even when participants have been food-deprived for up to 24 hours [9]. Modeling effects are also observed when the other person is not actually present. For example, Roth, Herman, Polivy, and Pliner [10] used a remote-confederate manipulation in which participants were exposed to a written indication of the food intake of 10 supposed previous participants. Even under those conditions, participants in the low-intake condition ate less than did participants in the high-intake condition. Furthermore, a direct comparison demonstrated that remote and live confederates are equally effective at influencing participants’ intake [11]. Thus, the effects of a social model on food intake are remarkably robust. The present research builds on this past work by examining the specific mechanisms through which social influences affect people’s food intake.

Social influences on food intake have been described from a normative perspective [1]. This account follows from the fact that the correct, appropriate, or acceptable amount to eat is, in many situations, fairly ambiguous. Imagine a cocktail party, a backyard barbeque, dinner at your grandmother’s house, or an all-you-can-eat buffet: How much should you eat in those situations? Can you go back for seconds or thirds? Must you go back for more, even if you are full? More often than not, one’s own internal signals (i.e., hunger and satiety) provide little or no clear guidance regarding how much one should eat; these signals are weak, often inordinately delayed, and sometimes absent altogether [12]. Thus, one may turn instead to the example provided by other people in the same situation. More specifically, it has been argued that social models provide a norm of appropriate food intake [1]; that is, people assume that if others eat a specific amount, then those others presumably know what they are doing and it is appropriate to follow their lead. Even if they do not know what they are doing, it may still be appropriate to eat the same amount as everyone else is eating. Normative influences have been shown to predict people’s healthy-eating intentions in the context of the theory of planned behavior [13], [14], and a normative explanation has also been used to account for the spread of obesity in social networks [15]. To date, however, no studies have directly examined the hypothesis that social models affect eating behavior by providing a norm of appropriate food intake.

Another important question arising from the modeling literature is whether or not people are aware of the influence of social cues on their food intake. The answer to this question has important implications for people’s ability to regulate their food intake. There is some preliminary evidence that people are not aware of the social influences on their food intake. For example, Vartanian, Herman, and Wansink [16] found that, although people’s food intake was strongly related to the eating behavior of their companion, they were more likely to report that hunger and the taste of the food were the most influential factors on their food intake. Furthermore, despite people’s assertions that hunger and taste were the primary determinants of how much they ate, the correlations between those factors and the amount that participants ate were quite small. Thus, not only do people seem to be unaware of social influences on their food intake, but they also overestimate the impact of other factors (especially hunger and the taste of the food).

### The Present Research

Social models are thought to influence people’s eating behavior by providing a norm of appropriate intake, but there is no direct evidence for this suggestion. Thus, the primary aim of this research was to directly test the hypothesis that the behavior of others affects perceived norms of appropriate food intake and, thereby, one’s actual food intake. Because most research on social influences has focused on women, the present studies included only female participants. Participants were exposed to either a low-intake model, a high-intake model, or no model (control condition). Experiments 1 and 2 used a remote-confederate manipulation and were conducted in the context of a cookie taste test. Experiment 3 used a live confederate and was conducted in the context of a task during which participants were given incidental access to food. We predicted that participants exposed to the low-intake model would eat less than would participants exposed to the high-intake model. We further predicted that the confederate’s intake would influence judgments of the appropriate amount to eat; specifically, participants exposed to the low-intake model would provide lower estimates of the appropriate amount to eat than would participants exposed to the high-intake model. Finally, we predicted that the perceived appropriateness norm would mediate the effects of the models’ intake on participants’ own food intake.

A secondary aim of this research was to examine people’s acknowledgement of social influences on their food intake. Prior studies have shown that people fail to acknowledge social influences on their food intake, but those studies are mostly correlational in nature. Using an experimental design allows us to examine acknowledgment of a factor that can be shown to causally influence eating behavior. We predicted that participants would rate the influence of other people’s behavior as a much less important determinant of their food intake than they would rate the taste of the food and how hungry they are. We also predicted that participants would be inaccurate in gauging the impact of other people’s behavior on their food intake.

## Experiment 1

### Method

#### Ethics statement

This study was approved by the Human Research Ethics Committee at the University of New South Wales. All participants provided written informed consent.

#### Participants

Participants were 78 female undergraduate students at an Australian university who received either $10 or course credit for their participation. Seven participants were excluded from the study (five due to suspicion about the manipulation, one for not following directions, and one because her food intake was more than 3 SD above the mean). Thus, the analyses below are based on 71 participants. Their mean age was 21.07 years (range = 18–28) and their mean body mass index (BMI; kg/m^2^) was 21.46 (range = 17.26–27.94). Participants’ ethnicity was not recorded.

#### Materials

*Remote confederate manipulation:* Participants in the experimental conditions were exposed to bogus information about the food intake of past participants via a sheet of paper that was “inadvertently” left visible. A clipboard taped to the top left corner of the table where participants’ completed the taste test was used to display this information. These sheets showed the cookie consumption of 10 alleged prior participants. Based on Roth et al. [10], the cookie consumption displayed in the low-intake condition ranged from 3 to 5 cookies (*M* = 4.00, *SD = *0.82) and the cookie consumption displayed in the high-intake condition ranged from 13 to 15 cookies (*M* = 14.00, *SD = *0.82). A control group was also included in which participants did not receive any information about the intake of past participants.

*Intake estimates:* As a manipulation check, participants were asked how many cookies other participants in the study had eaten, on average. Furthermore, so that we could compare their actual intake to their estimated intake, participants were asked to indicate how many cookies they themselves ate in the study.

*Appropriateness norms:* To assess the perceived appropriateness norm, participants indicated “how many cookies was an appropriate amount to eat in this situation.”

*Factors influencing food intake:* Participants completed a measure assessing their perceptions of the factors that influenced the amount that they ate during the experimental session [16]. These included “How hungry I was,” “How much I generally like the kind of food provided,” and “The taste of the food.” To assess participants’ acknowledgement of social cues, we included the item “How much other people in the study ate.” Each item was rated on a 7-point scale ranging from 1 (*Not at all an influence*) to 7 (*Very much an influence*).

#### Procedure

Participants signed up for a study on “hunger and taste perceptions” and were asked to refrain from eating for 3 hours prior to their experimental session. Participants were randomly assigned to the low-intake condition (*n* = 19), the high-intake condition (*n* = 26), or the control condition (*n* = 26). In the two remote-confederate conditions, the relevant information about past participants’ intake was already displayed when participants arrived for their session. After participants provided written consent, they reported when they last ate, and rated their current hunger level by placing a mark on a 10 cm line anchored by *Not at All Hungry* and *Extremely Hungry.* Participants were then provided with three types of cookies (chocolate chunk, triple chocolate, and Anzac [similar to an oatmeal cookie]) which they were asked to taste and rate on a variety of dimensions (e.g., how sweet, how salty). Each participant was given three pre-weighed bowls, each containing 50 freshly-baked mini cookies (each cookie was approximately 4.5 cm in diameter and weighed approximately 7 g). The experimenter emphasized the importance of providing accurate taste ratings and asked participants to have a sip of water in between cookie flavors in order to cleanse their palate. Participants were also told that they should feel free to help themselves to more cookies while waiting for the experimenter to return because any leftover cookies would have to be thrown out anyway.

For participants in the low-intake and high-intake conditions, the experimenter casually stated the following: “Don’t worry about adding your name to that sheet [motioning to the past-participant list]. We had been asking participants to write down how much they were eating so that we knew how much to order, but we’ve already ordered the food so you don’t need to add your name. You just need to fill out the three taste-rating forms.” All participants were then left alone for 10 minutes to make their taste ratings. After 10 minutes, the experimenter returned to remove the remaining cookies. The bowls were re-weighed and the number of cookies remaining was counted to determine the total number consumed.

Following the taste test, participants were given a questionnaire packet to complete. On the first page, they indicated how many cookies they ate in total and then completed the “factors influencing food intake” measure. Next, among several filler items, participants indicated how many cookies was an appropriate amount to eat, as well as how many cookies they thought other participants in the study had eaten. Participants then provided some basic demographic information, including their age, and height and weight (which were used to calculate their BMI). Finally, participants were probed for suspicion and were debriefed.

### Results

#### Manipulation check

A one-way ANOVA on participants’ estimates of how many cookies other participants in the study had eaten confirmed the effectiveness of the manipulation, *F*(2, 68) = 16.25, *p*<.001, η^2^_p_ = .32. Participants in the low-intake condition provided lower estimates of how much others had eaten (*M = *5.08, *SD* = 2.08) than did participants in the high-intake condition (*M = *12.50, *SD = *2.25), *p*<.001. Estimates for participants in the control condition (*M* = 10.06, *SD = *6.57) were significantly higher than the estimates provided by participants in the low-intake condition (*p* = .001) and were significantly lower than the estimates provided by participants in the high-intake condition (*p* = .05).

#### Cookie consumption

**Figure 1:**
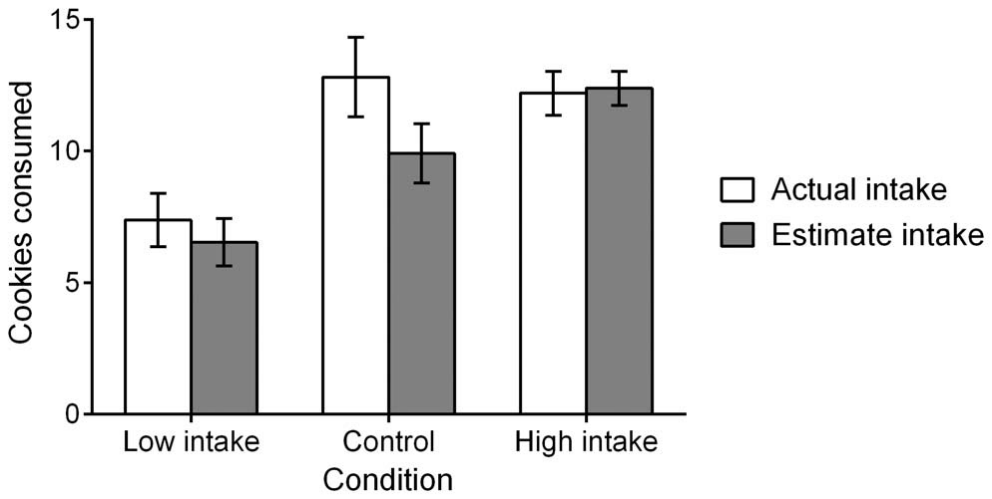
Actual and estimate cookie consumption in Experiment 1. Mean actual cookie consumption is displayed with white bars, and mean reported cookie consumption is displayed with gray bars. Error bars represent standard error of the mean.

Mean cookie consumption for each of the three groups is displayed in Figure 1 (white bars). A one-way ANOVA revealed that cookie consumption varied by condition, *F*(2, 68) = 5.47, *p* = .006, η^2^_p_ = .14. Participants in the low-intake condition ate significantly less than did participants in the high-intake condition, *p = *.003. Participants in the control condition ate significantly more than did participants in the low-intake condition (*p* = .008) but did not differ from participants in the high-intake condition (*p* = .71).

Participants’ estimates of their own intake are also varied by condition, *F*(2, 68) = 9.29, *p*<.001, η^2^_p_ = .22 (Figure 1, gray bars). Participants in the low-intake condition reported eating significantly less than did participants in the high-intake condition, *p*<.001. Participants in the control condition reported eating significantly more than did participants in the low-intake condition (*p* = .02) and significantly less than did participants in the high-intake condition (*p* = .05).

Further analyses using a repeated-measures ANOVA indicated that participants in the control condition significantly underestimated their intake (*M_diff_* = −2.90, *p*<.001), but that participants in the low-intake condition (*M_diff_* = −0.84, *p* = .35) and the high-intake condition (*M_diff_* = 0.19, *p* = .80) did not.

#### Appropriateness norms

**Figure 2:**
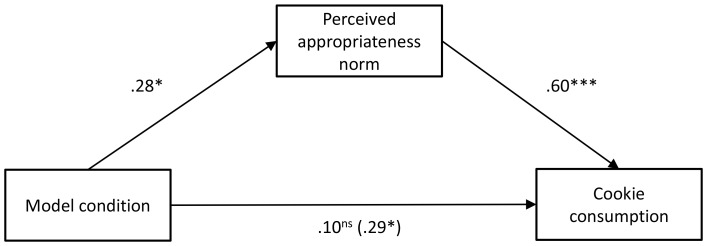
Mediation analysis for Experiment 1. All values are standardized beta coefficients. The number in parentheses represents the unmediated effect. *p<.05, **p<.01, ***p<.001.

Perceptions of how much was appropriate to eat also varied by condition, *F*(2, 65) = 3.20, *p* = .05, η^2^_p_ = .09. Planned contrasts indicated that participants in the low-intake condition (*M* = 5.92, *SD* = 4.36) reported a lower perceived norm of appropriate intake than did participants in the high-intake condition (*M* = 9.14, *SD* = 2.88), *p = *.02. Participants in the control condition (*M* = 8.58, *SD* = 5.34) reported a higher perceived norm of appropriate intake than did participants in the low-intake condition (*p* = .05) but did not differ from participants in the high-intake condition (*p* = .65) with respect to their perceived norm of appropriate intake. We next examined whether participants’ perceived norms about the appropriate amount to eat mediated the link between model condition and cookie consumption. Mediation analysis was conducted following the steps outlined by Baron and Kenny [17] and using the SPSS macro provided by Preacher and Hayes [18] (Figure 2): (1) model condition (−1 = low intake; 0 = control; 1 = high intake) was a significant predictor of cookie consumption; (2) model condition was significantly associated with perceived appropriateness norms (the proposed mediator); (3) perceived appropriateness norms was a significant predictor of cookie consumption when model condition was also included in the analysis; and (4) the direct effect of model condition on cookie consumption was no longer significant when perceived appropriateness norms was included in the analysis. Furthermore, the indirect path from model condition to cookie consumption through appropriateness norms was significant (95% CI = 0.40–2.61). Thus, perceived norms of appropriate intake mediated the link between model condition and participants’ food intake.

#### Rated influences on eating

A mixed model ANOVA was conducted to examine participants’ ratings of the extent to which their intake was influenced by how much others had eaten, compared to the taste of the food, how much they liked the food, and how hungry they were. Overall ratings of the importance of these factors did vary significantly, *F*(3, 204) = 59.66, *p*<.001, η^2^_p_ = .47. How much others ate was rated as a much less important influence (*M = *2.80, *SD = *2.02) than was taste (*M = *5.58, *SD = *1.14), liking (*M = *5.38, *SD = *1.47), and hunger (*M = *5.35, *SD = *1.39), *p*s <.001. Only ratings of the influence of other people’s behavior varied by model condition, *F*(2, 68) = 7.36, *p* = .001, η^2^_p_ = .18. Participants in the low-intake condition (*M* = 3.37, *SD* = 2.11) and participants in the high-intake condition (*M* = 3.50, *SD* = 2.16) rated other people’s behavior as more influential than did participants in the control condition (*M = *1.69, *SD = *1.23), *p = *.004 and *p = *.001, respectively; there was no difference between the low-intake and high-intake conditions, *p = *.82. Even for participants in the low-intake and high-intake conditions, how much others ate was rated as significantly less important than was taste, liking, or hunger, *p*s <.001.

Finally, to gauge participants’ accuracy in their reports of the extent to which they were influenced by social cues, we conducted a regression analysis predicting total cookies consumed from model condition, participants’ reports of the extent to which they were influenced by how much others ate (mean centered), and the interaction between these two factors. A significant positive beta coefficient for the interaction term would indicate that participants who reported being more influenced by the behavior of others were in fact more influenced by the social model. The interaction was not significant: *B* = 0.22, *SE* = 0.44, *p* = .61. Parallel analyses conducted with actual and self-reported influence of hunger (*B* = 0.09, *SE* = 0.21, *p* = .67), taste (*B* = 0.61, *SE* = 0.40, *p* = .13), and liking of the food (*B* = 0.53, *SE* = 0.30, *p* = .09) revealed the same pattern. Thus, participants were generally inaccurate in their evaluations of what factors influenced their food intake.

### Discussion

Experiment 1 found that social models (in this case remote confederates) had a significant impact on participants’ food intake, with participants eating less in the low-intake condition than in the high-intake condition. It has been proposed that social models influence eating behavior by establishing norms of appropriate intake [1], and our results provide the first direct support for this hypothesis. Participants’ perceptions of how much was appropriate to eat was less in the low-intake condition than in the high-intake condition, and perceived appropriateness norms mediated the effect of model condition on cookie consumption. Experiment 1 also examined the extent to which participants acknowledged the influence of social models on their food intake. Consistent with previous research [16], participants rated the behavior of others as significantly less influential than they rated the taste of the food, how much they liked the food, and how hungry they were. Furthermore, participants’ acknowledgement of social influences was unrelated to their actual susceptibility to the effects of the social model, suggesting that they may be unaware of the influence that the model has on their food intake.

In the Experiment 1, perceived norms of appropriate intake were assessed after participants had eaten because of our concern that specifying what was “appropriate” in advance might directly affect participants’ food intake. Experiment 2 replicated the first experiment, but participants were asked about the appropriateness norm either before they ate or after they ate.

## Experiment 2

### Method

#### Ethics statement

This study was approved by the Human Research Ethics Committee at the University of New South Wales. All participants provided written informed consent. The Australian National Statement on Ethical Conduct in Human Research recognizes mature minors as “young people who are mature enough to understand and consent, and are not vulnerable through immaturity in ways that warrant additional consent from a parent or guardian” (Section 4.2). Following those guidelines, no additional consent was obtained, beyond the standard informed written consent, from participants who were university students but who were also under the age of 18. This consent procedure has been approved by the ethics committee.

#### Participants

Participants were 126 female undergraduate students at an Australian university who received either $10 or course credit for their participation. Four participants were excluded from the study because their food intake was more than 3 SD above the mean. Thus, the analyses below are based on 122 participants. Their mean age was 19.80 years (range = 17–44) and their mean BMI was 20.96 (range = 16.00–43.21). (There was one extreme outlier in terms of BMI [BMI >40]. Removing this participant from the analyses had no impact on the results.) With respect to ethnicity, 26 were Caucasian, 73 were Asian, and 23 identified as “other.”

#### Procedure

As in Experiment 1, participants were randomly assigned to a low-intake condition (*n = *42), a high-intake condition (*n* = 41), or a control condition (*n* = 39). For half of the participants in each group, the question “how many cookies was an appropriate amount to eat in this situation” was asked with the “recent food intake” form that they completed just before they tasted the cookies (but after they were exposed to the previous participant list and after they had seen the bowls of cookies). For the remaining participants, the appropriateness norm question was asked after they finished eating (as in Experiment 1). The rest of the procedure was the same as that in Experiment 1.

### Results and Discussion

Participants’ estimates of how many cookies other participants in the study had eaten confirmed the effectiveness of the manipulation, *F*(1, 116) = 36.58, *p*<.001, η^2^_p_ = .39. Participants in the low-intake condition provided lower estimates of how much others had eaten (*M = *6.36, *SD* = 3.02) than did participants in the high-intake condition (*M = *11.60, *SD = *3.53), *p*<.001. Estimates for participants in the control condition (*M* = 7.04, *SD = *2.30) were significantly lower than the estimates provided by participants in the high-intake condition (*p*<.001) but did not differ from the estimates provided by participants in the low-intake condition (*p* = .34).

As in Experiment 1, there was a significant main effect of model condition on total cookies consumed, *F*(2, 116) = 4.11, *p* = .02, η^2^_p_ = .07: Participants in the low-intake condition (*M* = 7.69, *SD* = 2.98) ate significantly less than did participants in the high-intake condition (*M = *10.29, *SD = *4.17), *p = *.006. Participants in the control condition (*M = *9.59, *SD* = 5.19) ate significantly more than did participants in the low-intake condition (*p* = .05) but did not differ from participants in the high-intake condition (*p* = .44). The order of asking about perceived appropriateness norms had no impact on total cookies consumed (*p* = .57), and there was no order X model-condition interaction (*p* = .50). For perceptions of the appropriate amount to eat, there was a main effect of model condition, *F*(2, 113) = 5.78, *p* = .004, η^2^_p_ = .09. Planned contrasts indicated that participants in the low-intake condition (*M* = 6.03, *SD* = 3.82) reported a lower perceived norm of appropriate intake than did participants in the high-intake condition (*M* = 9.06, *SD* = 4.86), *p = *.001. Participants in the control condition (*M* = 6.76, *SD* = 3.60) reported a lower perceived norm of appropriate intake than did participants in the high-intake condition (*p* = .02) but did not differ from participants in the low-intake condition (*p* = .40) with respect to their perceived norm of appropriate intake. There was again no effect of order (*p = *.64) and no order X model-condition interaction (*p = *.39). Finally, replicating the findings of Experiment 1, perceived norms of appropriate intake mediated the link between model condition and participants’ food intake (95% CI = 0.16–0.86).

These findings indicate that, regardless of whether participants were asked about appropriateness norms before or after they ate, those in the low-intake condition ate less than did those in the high-intake condition, and also reported a lower perceived norm of appropriate intake. Furthermore, perceived appropriateness norms mediated the effect of model condition on cookie consumption, providing further evidence that appropriateness norms play a role in determining people’s food intake.

Experiments 1 and 2 used a remote confederate manipulation in the context of a taste test, and it is possible that the specific features of this experimental design influenced the results. First, in the remote confederate manipulation, participants are provided with concrete information about how much others ate from the beginning of the experiment. This type of exposure does not parallel a real-world social eating situation in which one must pay attention to, and keep track of, how much others are eating, with the final intake amount becoming apparent only at the end of the eating occasion. Second, the taste-test context leaves ambiguous the meaning of the term “appropriate”; that is, should appropriateness be judged in relation to the taste test or in relation to the social situation? Experiment 3 addressed these issues by using a live confederate rather than a remote confederate, and by giving participants incidental access to snack foods while completing an unrelated task rather than using the taste-test paradigm.

## Experiment 3

### Method

#### Ethics statement

This study was approved by the Human Research Ethics Committee at the University of New South Wales. All participants provided written informed consent. The Australian National Statement on Ethical Conduct in Human Research recognizes mature minors as “young people who are mature enough to understand and consent, and are not vulnerable through immaturity in ways that warrant additional consent from a parent or guardian” (Section 4.2). Following those guidelines, no additional consent was obtained, beyond the standard informed written consent, from participants who were university students but who were also under the age of 18. This consent procedure has been approved by the ethics committee.

#### Participants

Participants were 94 female undergraduates at an Australian university who received $15 or course credit for their participation. One participant was excluded because her food intake was more than 3 SD above the mean. An additional 26 participants had initially taken part in the study but did not eat anything during the task. Because the distribution of these participants across conditions was uneven (low intake = 4; control = 14; high intake = 8), and because it is not clear why they did not eat (e.g., perhaps they were uncomfortable in the experimental setting), these participants were excluded from the analyses. The results were the same when those participants were included in the analyses, but the interpretation of the means is more ambiguous given the issues just noted. Thus, we consider it more appropriate to interpret the data without these participants. Thus, the final sample consisted of 93 individuals. Their mean age was 20.52 years (range = 17–26) and their mean BMI was 21.91 (range = 16.97–41.42). (There was one extreme outlier in terms of BMI [BMI >40]. Removing this participant from the analyses had no impact on the results.) With respect to ethnicity, 27 were Caucasian, 54 were Asian, and 12 identified as “other.”

#### Confederate manipulation

Participants completed a murder-mystery task in which they were given 15 minutes to read through evidence and transcripts related to a murder case and to determine who the guilty party was. Participants in the experimental conditions took part in the study at the same time as another participant (actually a female experimental confederate). Four different women served as confederates, counterbalanced across conditions, and all were university aged and had a BMI in the healthy range. Participants and confederates were seated across from one another at a table, but worked on the murder-mystery task individually and were instructed not to discuss the task with one another. Participants in the control condition completed the study alone. All participants and confederates were given their own bowl of M&Ms and a glass of water, which they were told they could help themselves to while completing the murder-mystery task. Each participant was provided with 152 grams of M&Ms (each M&M weighed approximately 1 g). In the low-intake condition, the confederate was instructed to eat 2 M&Ms (one at the start of the task and a second one halfway through the task). In the high-intake condition, the confederate was instructed to eat approximately 35 M&Ms. In order to approximate this number, a clock was visible behind the participant’s shoulder and the confederate was instructed to eat one M&M approximately every 20–30 seconds (*M* = 33.97, *SD* = 1.50).

#### Measures

*Intake estimates:* As a manipulation check, participants in the experimental conditions were asked to indicate how many M&Ms the other participant (i.e., the confederate) ate. So that we could compare their actual intake to their estimated intake, participants were also asked to indicate how many M&Ms they themselves ate.

*Assessing perceived norms:* To assess the perceived appropriateness norm, participants were asked to indicate how many M&Ms was an appropriate amount to eat in this situation. Furthermore, in order to rule out the possibility that the perceived appropriateness norm merely reflects participants’ perceptions of how others would behave (a simple descriptive norm) [19], [20], participants were also asked to estimate how many M&Ms other participants in the study had eaten, on average. Because participants were exposed to only one confederate (unlike in Experiments 1 and 2), determining how much other participants in general had eaten would have to be an inference.

*Factors influencing food intake:* Participants completed a 15-item measure assessing their perceptions of the factors that influenced the amount that they ate during the experimental session, including “How hungry I was,” “How much I generally like the kind of food provided,” and “How much the other person ate [control condition: How much other people in the study ate]”. Each item was rated on a 7-point scale ranging from 1 (*Not at all an influence*) to 7 (*Very much an influence*).

#### Procedure

Participants signed up for a study on “problem-solving processes” and were randomly assigned to one of three conditions prior to their arrival for the experiment (low-intake condition, *n* = 31; high-intake condition, *n* = 30; control condition, *n* = 33). After participants provided written consent, the experimenter introduced the murder-mystery task, and then left participants (and confederates) alone for 15 minutes to complete the task during which time they had access to the M&Ms. When the experimenter returned, she gave participants a questionnaire packet to complete, removed the M&Ms, and, in the experimental conditions, asked the confederate to come with her to a different room. Participants were asked to indicate how much they ate and also to report on the factors that influenced their food intake. Next, participants in the experimental conditions indicated how much the confederate ate. All participants then reported how much was appropriate to eat and how much other participants in the study had eaten, on average. Finally, participants provided some basic demographic information (age, height and weight, ethnicity). After completing the questionnaire packet, participants were probed for suspicion–no participant guessed the purpose of the study–and were debriefed. After participants left, the bowl of M&Ms was reweighed to determine the amount eaten.

### Results

#### Manipulation check

Participants’ estimates of how many M&Ms the confederate ate confirmed the effectiveness of the manipulation, *F*(1, 54) = 60.71, *p*<.001, η^2^_p_ = .53. Participants in the low-intake condition reported that the confederate had eaten, on average, 4.48 M&Ms (*SD* = 3.88) whereas participants in the high-intake condition reported that the confederate had eaten, on average, 17.78 M&Ms (*SD = *8.27).

#### M&M consumption

**Figure 3:**
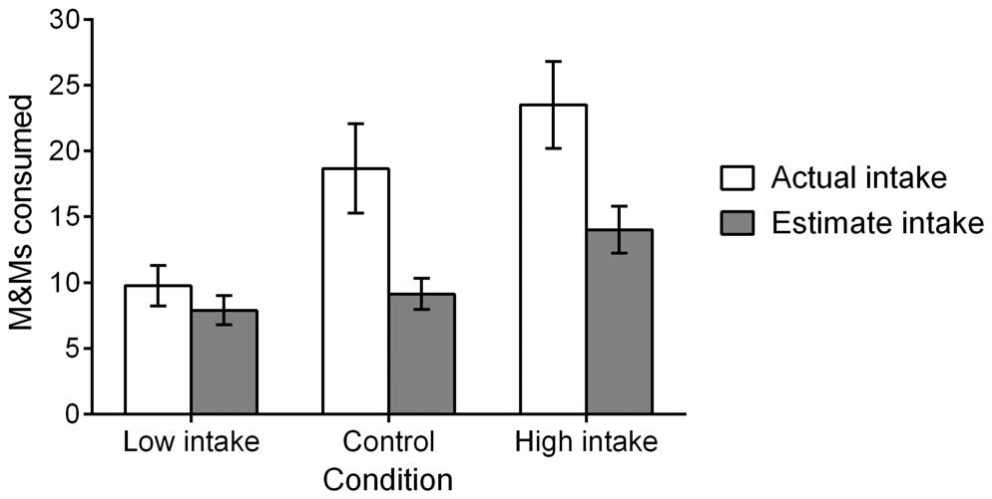
Actual and estimate cookie consumption in Experiment 3. Mean actual cookie consumption is displayed with white bars, and mean reported cookie consumption is displayed with gray bars. Error bars represent standard error of the mean.

Mean M&M consumption for each of the three groups is displayed in Figure 3 (white bars). A one-way ANOVA revealed that M&M consumption varied by condition, *F*(2, 90) = 5.75, *p* = .004, η^2^_p_ = .11. Participants in the low-intake condition ate significantly less than did participants in the high-intake condition, *p = *.001. Participants in the control condition ate significantly more than did participants in the low-intake condition (*p* = .03) but did not differ from participants in the high-intake condition (*p* = .24). Including the non-eaters in this analysis did not change the outcome: *F*(2, 116) = 3.48, *p* = .03, η_p_^2^ = .06.

Participants’ estimated intake revealed that reported M&M consumption varied by condition, *F*(2, 86) = 5.08, *p* = .008, η^2^_p_ = .11 (Figure 3, gray bars). Participants in the low-intake condition reported eating significantly less than did participants in the high-intake condition, *p = *.003. Participants in the control condition did not report eating significantly more than did participants in the low-intake condition (*p* = .53) but reported eating significantly less than did participants in the high-intake condition (*p* = .02).

Further analyses using a repeated-measures ANOVA indicated that participants in the control condition significantly underestimated their intake (*M_diff_* = −8.76, *p*<.001), as did participants in the high-intake condition (*M_diff_* = −9.43, *p*<.001), but participants in the low-intake condition (*M_diff_* = −1.70, *p* = .45) did not.

#### Appropriateness norms

Perceptions of how much was appropriate to eat also varied by condition, *F*(2, 80) = 3.13, *p* = .05, η^2^_p_ = .07. Participants in the low-intake condition (*M* = 8.26, *SD* = 5.51) reported a lower perceived norm of appropriate intake than did participants in the high-intake condition (*M* = 12.93, *SD* = 8.08), *p = *.02. Participants in the control condition (*M = *10.23, *SD = *6.97) did not differ from either the low-intake condition (*p = *.30) or the high-intake condition (*p* = .15). Including the non-eaters in this analysis did not change the outcome: *F*(2, 106) = 3.30, *p* = .04, η_p_^2^ = .06.

Parallel findings were observed for perceptions of how much other participants in the study had eaten (the descriptive norm), *F*(2, 86) = 19.27, *p*<.001, η^2^_p_ = .31. Participants in the low-intake condition (*M* = 6.72, *SD* = 3.82) reported a lower perceived descriptive norm than did participants in the high-intake condition (*M* = 16.80, *SD* = 9.55), *p*<.001. Participants in the control condition (*M* = 8.33, *SD* = 5.37) did not differ from participants in the low-intake condition (*p* = .36) in terms of their perceived descriptive norm, but reported a lower descriptive norm than did participants in the high-intake condition (*p*<.001).

**Figure 4:**
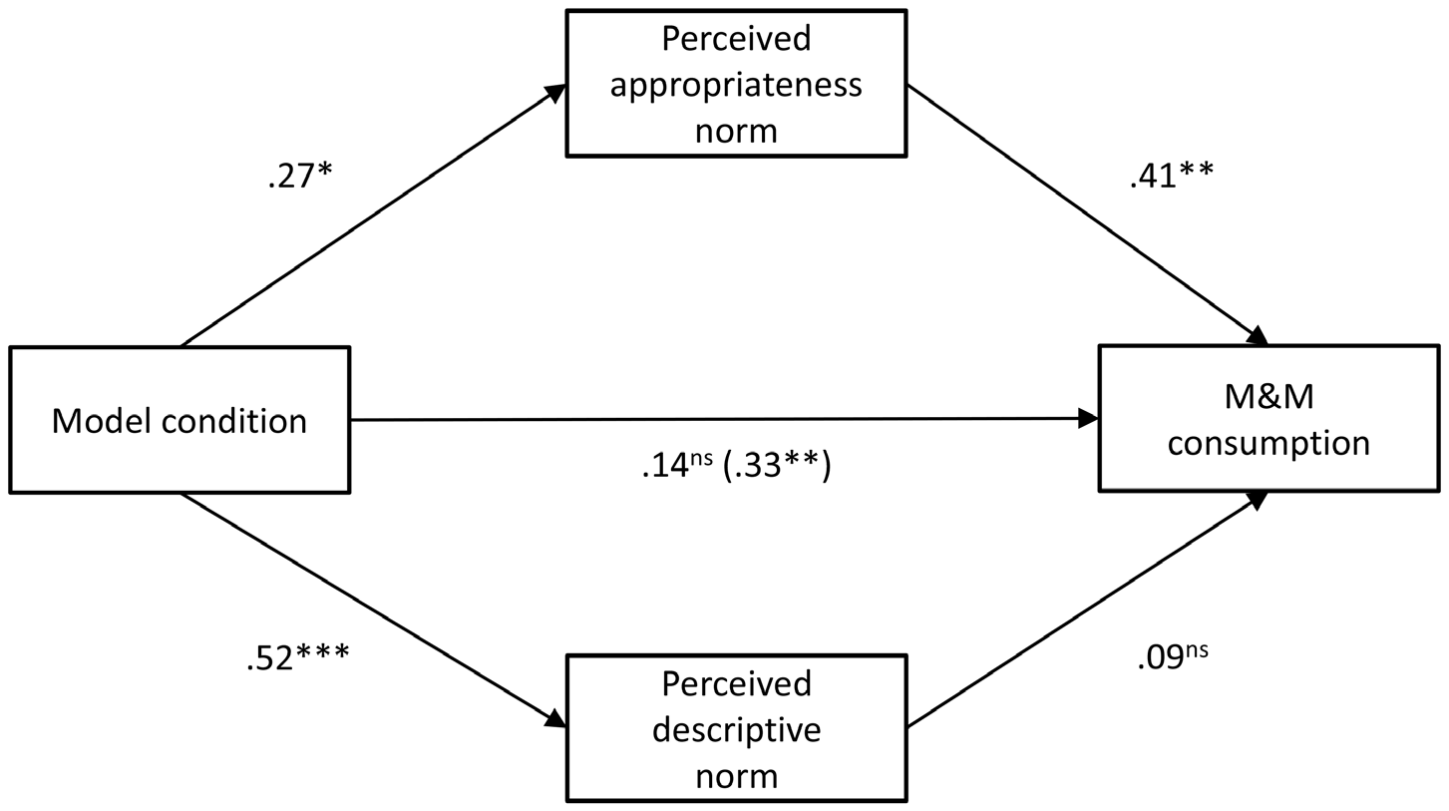
Multiple mediation analysis for Experiment 3. All values are standardized beta coefficients. The number in parentheses represents the unmediated effect. *p<.05, **p<.01, ***p<.001.

We next conducted a multiple mediation analysis (18) to test the indirect effects of the appropriateness and descriptive norms. Those two items were significantly correlated with one another (*r* = .67, *p*<.001). When both mediators were entered simultaneously, appropriateness norms was a significant mediator of the effect of model condition on M&M intake (95% CI: 0.59–5.68), but the descriptive norm was not (95% CI: −2.44–4.49) (see Figure 4 for path coefficients). Including the non-eaters in this analysis did not change the pattern of results, but did somewhat attenuate the indirect effects: The 95% CI for appropriateness norms was −0.42–4.03, and the 95% CI for the descriptive norm was −3.98–4.04.

#### Rated influences on eating

A mixed model ANOVA was conducted to examine participants’ ratings of the extent to which their intake was influenced by how much the confederate/other people in the study had eaten, compared to how much they liked the food and how hungry they were. Overall ratings of the importance of these factors did vary significantly, *F*(2, 180) = 48.26, *p*<.001, η^2^_p_ = .35. The behavior of others (*M = *2.95, *SD = *2.06) was rated as less of an influence than was liking of the food (*M* = 5.42, *SD = *1.64; *p*<.001), but did not differ from ratings for hunger (*M = *3.32, *SD = *2.12; *p* = .23). Only ratings of the influence of the behavior of others varied by condition, *F*(2, 90) = 7.35, *p* = .001, η^2^_p_ = .14. Participants in the low-intake condition (*M* = 3.10, *SD* = 2.07) and participants in the high-intake condition (*M* = 3.83, *SD* = 1.97) rated the behavior of others as more influential than did participants in the control condition (*M = *1.97, *SD = *1.75), *p = *.023 and *p*<.001, respectively; there was no difference between the low-intake and high-intake conditions, *p = *.14. Even for participants in the low-intake and high-intake conditions, the behavior of others was rated as significantly less important than liking of the food (*p*s ≤.001), and again did not differ from hunger (*p*s >.22).

Finally, to gauge the accuracy of participants’ self-reports, we conducted a regression analysis predicting participants’ M&M intake from model condition, their self-reports of how much they were influenced by how much others ate (mean centered), and the interaction between these two factors. A significant positive interaction term would indicate a degree of accuracy in participants’ self-reports. The interaction was not significant: *B* = 2.21, *SE* = 4.17, *p* = .60. A parallel analysis conducted with actual and self-reported influence of the liking of the food (*B* = 0.65, *SE* = 0.65, *p* = .32) revealed the same pattern. Thus, as in Experiment 1, participants were generally inaccurate in their evaluations of what factors influenced their food intake.

### Discussion

As in Experiments 1 and 2, participants in the low-intake condition ate less than did participants in the high-intake condition, and also reported a lower perceived norm of appropriate intake than did participants in the high-intake condition. Also replicating the findings of Experiments 1 and 2, Experiment 3 found that norms of appropriate intake mediated the effect of the confederate’s intake on participants’ food intake. Finally, the other person’s behavior was rated as a less important influence on participants’ food intake than was their liking of the food, despite the strong influence of others on their behavior.

## General Discussion

Social models can act as a powerful influence on people’s food intake. Consistent with previous research [1], we found that participants ate less in the presence of social models who ate very little than they did in the presence of social models who ate a large amount. One of the primary aims of this study was to test the hypothesis that social models influence food intake by providing a norm of appropriate intake. In all three experiments, participants in the low-intake condition indeed reported a lower perceived norm of the appropriate amount to eat than did participants in the high-intake condition. We also found that norms of appropriate intake fully mediated the link between model condition and participants’ food intake. Experiment 3 further distinguished between an appropriateness norm and a more general descriptive norm (i.e., perceptions of how others generally behave). Multiple mediation analysis showed that the appropriateness norm, but not the simple descriptive norm, mediated the link between model condition and participants’ food intake. These findings offer the first direct support for the hypothesis that social models influence food intake by providing a norm of appropriate eating behavior.

Our results also provide a number of interesting insights into the nature of the modeling of food intake. First, the pattern of results across studies was remarkably similar, despite the fact that in two of the experiments (Experiments 1 and 2) the social model was not actually present. From a methodological standpoint, these findings are important because they indicate that the remote-confederate manipulation is a viable and cost effective option for conducting research on modeling of food intake [11]. Theoretically, the parallel results emerging from these two manipulations suggest that information about the appropriate amount to eat can be derived in different ways. In the case of a live model, the participant is required to attend to and keep track of the model’s food intake in real time in order to arrive at an estimate of the appropriate amount to eat. However, this process is absent in the remote-confederate context, indicating that the real-time tracking of the model’s intake is not a necessary condition for modeling to take place. Although live models and remote models might both provide information about what is an appropriate amount to eat, it may be the case that certain additional processes, such as ingratiation [5], [21] or mimicry [22], are involved when eating in the presence of others that are not operating in the remote-confederate situation. Furthermore, it is also possible that the simple descriptive norm (information about what others have done) plays a more important role in the remote-confederate situation whereas the perceived appropriateness norm plays a more important role in the live-confederate situation. Identifying the overlapping and non-overlapping mechanisms across these two experimental approaches would contribute to a more thorough understanding of the modeling of food intake.

Second, our findings suggest that social models may be more likely to inhibit than to augment intake. In all three experiments, participants in the low-intake condition ate significantly less than did participants in the control condition, but participants in the high-intake condition did not eat significantly more than did participants in the control condition. Thus, low-intake models appear to inhibit intake, but high-intake models do not appear to augment intake. These findings are consistent with the notion that people are generally motivated to maximize their intake of palatable food without appearing to eat “excessively” [1]. Low-intake confederates suppress eating by placing a relatively low ceiling on appropriate intake, whereas the high-intake confederate appears to give participants permission to eat as much as they want without feeling as though they have eaten “too much.” In all three experiments, participants in the low-intake condition did, on average, eat more than the low-intake confederates. However, it seems likely that the amount consumed in each case was still low enough that there was no real risk of being seen as eating “too much.”

An alternative explanation for the current findings might be that providing participants with a large portion of food in the control condition creates a high-consumption norm that inflates intake in that condition. This seems unlikely for a number of reasons: First, the amount of food provided to participants is the same in each condition. Second, the same pattern of results emerged in all three experiments, regardless of whether the context was a taste test (in which an underlying norm to eat a lot might be particularly strong) or a context in which participants had incidental access to food. Third, in Experiment 3 (involving incidental access to food), the amount eaten in the control condition was not particularly high in absolute terms (<100 kcal), and that condition also had the greatest number of non-eaters. Fourth, the high-intake confederate in each experiment ate only a small portion of the total amount of food provided. If portion size was dictating the intake norm for participants in the control condition, then even the high-intake confederate should have functioned as an “inhibitory” model and participants should have eaten less in the high-intake condition than in the control condition, which was not the case. Thus, the inhibitory power of the low-intake model seems to provide a better account of the findings of our experiments.

The motivation to avoid eating excessively can also potentially explain our findings regarding participants’ accuracy in estimating how much they ate. Specifically, we suggest that participants will underreport their intake when they have eaten more than they believe others have eaten. Indeed, participants in the control condition in Experiment 1 (actual-own-intake minus estimated-others’-intake = +2.90, *p* = .002) underreported their own intake, as did participants in the control condition in Experiment 3 (actual-own-intake minus estimated-others’-intake = +10.90, *p*<.001), and the high-intake condition in Experiment 3 (actual-own-intake minus estimated-others’-intake = +6.70, *p* = .02). Participants in the high-intake condition in Experiment 1 did not eat more than they thought others ate (actual-own-intake minus estimated-others’-intake = −0.27, *p* = .76), and these participants also accurately reported their intake. Note, however, that this interpretation does not apply to participants in the low-intake conditions. As noted above, because their overall intake was relatively low, they were not in danger of eating “excessively” and so no underreporting was necessary, even if they did eat more than what others ate (Experiment 1, actual-own-intake minus estimated-others’-intake = +2.16, *p* = .04; Experiment 3, actual-own-intake minus estimated-others’-intake = +3.45, *p* = .22). This explanation, of course, is entirely *ad hoc* and future research will be needed to provide more direct support for this hypothesis.

### Awareness of Social Influences

The present studies provide further evidence that people do not acknowledge the impact of other people’s behavior on their food intake. Consistent with Vartanian et al. [16], participants rated the impact of other people’s behavior lower than they did the impact of how hungry they were (Experiment 1) and how much they liked the food (Experiments 1 and 3). Thus, participants are generally less likely to acknowledge the influence of social factors on their food intake compared to other common-sense factors such as hunger and the taste of the food. Still, participants in the experimental conditions (i.e., those exposed to social cues) were more likely to report that they were influenced by the behavior of other people than were participants in the control condition (i.e., those not exposed to any social cues). These findings suggest that participants might at least acknowledge to a limited extent the influence of social cues on their food intake. Furthermore, participants appear to be somewhat sensitive to situational variations with respect to their explanations for their food intake. Specifically, hunger (along with taste) was highly rated as a reason for eating cookies in Experiment 1, but hunger was rated lower than taste (and no different from the behavior of others) as a reason for eating M&Ms in Experiment 3. These attributions seem sensible because hunger might well be more relevant to cookie consumption than to M&M consumption (because the former may be more filling). Although these two sets of findings suggest some sensitivity in participants’ explanations for their eating behavior, many participants showed no evidence of accuracy in their reports of what influenced their food intake. Specifically, participants who acknowledged being influenced by the behavior of others were no more likely to be influenced by the social model than were participants who denied being influenced by the behavior of others. The same was true for ratings of the influence of hunger and taste. Thus, most participants failed to accurately report on when and how various factors influence their food intake. Of course, failure to accurately report on the factors influencing one’s food intake does not necessarily reflect a lack of awareness–instead, it might reflect an unwillingness to admit to those influences, as some recent research suggests [23].

### Limitations and Future Directions

One limitation of the present research is that our samples consisted exclusively of women. Past research on modeling of food intake among men has produced mixed results [4], [24], and it may be that men are less concerned than are women about behaving appropriately in the domain of eating (cf. [25]). Thus it remains an open question whether the same processes identified in our studies would be observed among men. It would also be interesting to explore the conditions under which social models will and will not convey norms of appropriate intake. For example, some models might not be seen as relevant guides of appropriate behavior–Cruwys et al. [26] found that female students modeled the intake of another female from their own university, but not a female from a different university, presumably because the outgroup member was not seen as a relevant model. Furthermore, estimates of appropriate intake might be adjusted based on the characteristics of the model–McFerran, Dahl, Fitzsimons, and Morales [27] found that participants modeled the behavior of a thin confederate to a greater extent than they did the behavior of an obese confederate. It is also possible that individual or situational differences in the desire to behave “appropriately” would moderate the effect of a social model [28].

## Conclusion

Our findings suggest that people model the eating of others in ambiguous eating situations at least in part because those others provide information about the appropriate amount to eat. When others eat sparingly, so do the observers, whereas when the models eat a large amount, the observers feel free to eat as much as they want (which did not differ from the amount eaten by those eating alone in the absence of a model). Despite this influence of the model, people may be unaware that their behavior is so strongly affected by what others do. The implications for the current obesity epidemic are that, if societal norms have loosened so that in most situations people feel free to eat as much as they want, then combining those lax social norms with increased availability of food and larger portion sizes may increase intake overall. Future research might focus on determining whether manipulating appropriateness norms can help to reduce overeating and thus weight gain and obesity.
